# Indwelling pleural catheters for malignancy-associated pleural effusion: report on a single centre’s ten years of experience

**DOI:** 10.1186/s12890-019-1002-8

**Published:** 2019-12-02

**Authors:** Nikolaj Frost, Martin Brünger, Christoph Ruwwe-Glösenkamp, Matthias Raspe, Antje Tessmer, Bettina Temmesfeld-Wollbrück, Dirk Schürmann, Norbert Suttorp, Martin Witzenrath

**Affiliations:** 1 Department of Infectious Diseases and Pulmonary Medicine, Charité – Universitätsmedizin Berlin, corporate member of Freie Universität Berlin, Humboldt-Universität zu Berlin, and Berlin Institute of Health, Augustenburger Platz 1, D-13353 Berlin, Germany; 2 Institute of Medical Sociology and Rehabilitation Science, Charité – Universitätsmedizin Berlin, corporate member of Freie Universität Berlin, Humboldt-Universität zu Berlin, and Berlin Institute of Health, Berlin, Germany; 3 Klinik für Pneumologie – Evangelische Lungenklinik Berlin Buch, Berlin, Germany; 4 Division of Pulmonary Inflammation, Charité – Universitätsmedizin Berlin, corporate member of Freie Universität Berlin, Humboldt-Universität zu Berlin, and Berlin Institute of Health, Berlin, Germany

**Keywords:** Malignant pleural effusion, Indwelling pleural catheter, Palliative care, Pleurodesis

## Abstract

**Introduction:**

Recurrent pleural effusion is a common cause of dyspnoea, cough and chest pain during the course of malignant diseases. Chemical pleurodesis had been the only definitive treatment option until two decades ago. Indwelling pleural catheters (IPC) emerged as an alternative, not only assuring immediate symptom relief but also potentially leading to pleurodesis in the absence of sclerosing agents.

**Methods:**

In this single-centre retrospective observational study patient characteristics, procedural variables and outcome in a large population of patients with IPC in malignancy were evaluated and prognostic factors for pleurodesis were identified.

**Results:**

From 2006 to 2016, 395 patients received 448 IPC, of whom 121 (30.6%) had ovarian, 91 (23.0%) lung and 45 (11.4%) breast cancer. The median length of IPC remaining in place was 1.2 months (IQR, 0.5–2.6), the median survival time after insertion 2.0 months (IQR, 0.6–6.4). An adequate symptom relief was achieved in 94.9% of all patients, with no need for subsequent interventions until last visit or death. In patients surviving ≥30 days after IPC insertion, pleurodesis was observed in 44.5% and was more common in patients < 60 years (HR, 1.72; 95% CI, 1.05–2.78; *p* = 0.03). The use of an additional talc slurry via the IPC was highly predictive for pleurodesis (HR 6.68; 95% CI, 1.44–31.08; *p* = 0.02). Complications occurred in 13.4% of all procedures (*n* = 60), 41.8% concerning infections (local infections at the tunnel/exit site (*n* = 14) and empyema (*n* = 11)), and 98.3% being low or mild grade (*n* = 59). Complication rates were higher in men than women (18.6 vs. 12.4%, *p* = 0.023).

**Conclusion:**

High efficacy in symptom relief and a favourable safety profile confirm IPC as suitable first line option in most malignant pleural effusions. The study presents the largest dataset on IPC in gynaecologic cancer to date. Gender-specific differences in complication rates warrant further study.

## Background

Recurrent pleural effusion is a common cause of dyspnoea, cough and chest pain in malignant diseases. Approximately 15% of these patients develop malignant pleural effusion (MPE) during the course of their disease [1]. Survival is very limited with an average of 5.5 months in lung cancer, 13 months in breast cancer and 24 months in ovarian cancer [2–4]. In contrast, paramalignant pleural effusion is disease-associated, though not caused by malignant cells directly infiltrating the pleura, but rather resulting from lymphoid or bronchial obstruction, infiltration of the thoracic duct (leading to chylothorax), cachexia or the treatment itself [5, 6]. For slowly regenerating effusions, repeated thoracenteses might be justified, but in general, a definitive procedure should be pursued [7]. Chemical pleurodesis, especially with talc (TP), has been the sole definitive treatment option for decades. Efficacy rates between 60 and 90% have been reported, but the longer patients survive the more pleurodesis failures occur [8–10]. Further, TP may be associated with severe complications, particularly acute respiratory failure. Lastly, the procedure is not feasible in cases of lung entrapment, e.g. in parenchymal restriction due to bronchial obstruction.

Indwelling pleural catheters (IPC) emerged as an alternative two decades ago. The catheter is placed percutaneously, and the procedure can be performed in an outpatient setting [11]. Although its primary objective is symptom relief via repeated drainage, pleurodesis in the absence of any sclerosing agent occurs in approximately 45% of the cases [12]. A Cochrane network meta-analysis from 2016 stated that IPC confer highly clinically relevant benefits for patients, making them an appropriate first-line treatment option [13]. Beyond that, IPC offer a satisfying option for patients not suitable for TP due to lung entrapment. Third, IPC have a favourable safety profile, as most of the rare complications can easily be managed [14, 15]. Last, not pleurodesis itself, but a durable symptom relief and patient comfort should be the main goals in these patients [7, 16]. In comparison with TP, the time spent periprocedurally in hospital is much shorter with IPC [17]. In the TIME2 trial, use of an IPC was associated with higher readmission rates due to procedure-related complications. However, patients spent substantially less time in hospital in the year after the procedure as compared to those with TP [18]. These results were confirmed in the multicentre Australasian Malignant Pleural Effusion (AMPLE) Trial and the Dutch NVALT-14 trial [19, 20]. Finally, IPC and a subsequent TP via the catheter may also be combined, as shown in the IPC-PLUS trial [21]. For these reasons IPC are increasingly used and have partially replaced TP in the setting of MPE.

The purpose of the present study was to evaluate clinical outcomes with IPC in the general setting of malignancy. Secondary goals were to assess survival outcomes depending on patient and clinical variables and to determine predictors of pleurodesis, thus helping physicians to better guide clinical care.

## Methods

For this retrospective single centre study, patients with an underlying malignancy and having received an IPC due to symptomatic recurrent pleural effusion treated at the Department of Infectious Diseases and Pulmonary Medicine at the Charité – Universitätsmedizin Berlin were identified by using a departmental database and the hospital’s clinical reporting system. Approval for the study was obtained from the Charité – Universitätsmedizin Berlin ethics committee (EA2/037/18). Data on patients’ baseline demographics, tumour entity, aetiology of pleural effusion, catheter laterality, pleurodesis, complications, time of catheter removal, need for subsequent procedures and day of last follow-up or death were collected. MPE had to be proven by cytology, whereas paramalignant and cytology-negative pleural effusions were diagnosed in the absence of radiologic suspicion of pleural carcinosis and repeated (*n* ≥ 2) negative fluid cytology.

Pre-interventional pleuroscopies or pleural biopsies were not routinely performed. All catheters (PleurX®, CareFusion, San Diego (CA), USA) were placed in the endoscopy unit, ultrasound-guided and under local anaesthesia by a pulmonologist. Prophylactic peri-interventional antibiotics were not routinely administered. Every patient was revisited at least once the day after IPC placement in the endoscopy unit. Outpatient drainage was performed by either a specialized ambulatory care service or the patients themselves. According to the local standard of care, gravity bags were employed rather than vacuum bottles. They are mainly used in Germany assuring a slow and comfortable drainage. Patients were instructed to connect a gravity bag daily until the fluid accumulation decreased to less than 200 ml in 24 h, then the interval was extended to a two-day regimen. Pleurodesis was assumed in the presence of less than 200 ml of pleural effusion per week and was defined as successful if no further intervention was needed after catheter removal. All complications were graded using the Clavien-Dindo classification for surgical complications. Thus, grade I/II complications only require pharmacologic, grade III surgical, endoscopic or radiological interventions. Grade IV is life-threatening, grade V denotes a procedure-related death [22].

Follow-up data were obtained from the department’s clinical database, from practitioners and from the Charité hospital tumour documentation system. Loss to follow-up was defined as missing data ≥30 days between last documented visit and death.

### Statistical analysis

Demographics and disease data were described and compared using the Pearson Chi^2^-test, Fisher’s exact test or Mann-Whitney-U test, according to the level of measurement. Binary logistic regression analyses were used to identify factors associated with pleurodesis. Overall survival was assessed with the Kaplan-Meier method in two different ways: OS1 was defined as the interval in months between diagnosis of the malignancy and death, OS2 as the interval in months between IPC insertion and death. *P*-values comparing survival curves were calculated with log-rank tests. Univariate Cox regression analysis was performed on a set of baseline patients, tumour and treatment characteristics to identify predictors of survival. Concerning pleurodesis and complications, Cox analysis with a time-dependent covariate was performed to exclude immortal time bias. Furthermore, multivariate Cox regression was applied to the explanatory variables with statistical significance in univariate analysis. All analyses were performed using IBM SPSS statistics version 24 (IBM, Armonk, NY, USA). A *p*-value < 0.05 (two-tailed) was defined as statistically significant.

## Results

Between 2006 and 2016, 395 patients received 448 IPC. In contrast to other published case series, the majority of patients suffered from ovarian cancer (*n* = 121, 30.6%). Baseline demographics are shown in Table 1. Supply with an IPC was preceded by either at least two thoracenteses or a chest tube in 275 procedures (61.3%). In 46 cases (10.3%), an IPC was used following TP failure. 53 patients (13.4%) received bilateral IPC with a median delay between placements of 7 days (range 1–562, IQR 3.8–48.5). Per definition, IPC had to be in-situ simultaneously. Bilateral effusions were significantly correlated to patients with ovarian cancer (19.8% vs. 10.6% in the remaining entities, *p* = 0.002). MPE accounted for 346 effusions (77.2%), with the highest percentage in breast cancer followed by ovarian and lung cancer (100 vs. 92.1 vs. 85.9%, *p* = 0.03). The other causes were disease-associated but cytology-negative effusions (*n* = 71, 15.8%), postobstructive paramalignant effusions (*n* = 26, 5.8%) or were treatment-related (*n* = 5, 1.1%). The probability for paramalignant effusions was highest in thoracic malignancies (lung cancer vs. else, *p* = 0.002).

**Table 1:** Baseline characteristics

Variable	n	%
Patients		
Total	395	100.0
Male	130	32.9
Female	265	67.1
Age (years)^a^	65	15–92
Malignancy		
Ovarian cancer	121	30.6
Lung cancer	91	23.0
Breast cancer	45	11.4
GI neoplasia^b^	31	7.8
Hepatobiliary and pancreatic cancer^c^	27	6.8
Hematologic neoplasia^d^	22	5.6
Other gynaecologic neoplasia^e^	15	3.8
Sarcoma	12	3.0
Head and neck cancer	6	1.5
Other^f^	25	6.3
Catheters		
Total	448	
Laterality		
Left	151	38.2
Right	194	49.1
Bilateral	50	12.7
Pleural Effusion		
Malignant	346	77.2
Disease-associated, cytology negative	71	15.8
Cachexia/Hypoalbuminemia	35	7.8
Unknown aetiology	36	8.0
Paramalignant (Postobstructive)	26	5.8
Bronchi	18	4.0
Lymphoid vessels	1	0.2
Chylothorax	7	1.6
Treatment associated	5	1.1
Drug related (Dasatinib)	2	0.4
Surgery related	2	0.4
Radiofrequency ablation	1	0.2

^a^ Values are given as “median (range)”

^b^ Stomach cancer: *n* = 14, colorectal cancer: *n* = 8, oesophageal cancer: *n* = 7, anal cancer: *n* = 2

^c^ Hepatocellular cancer: *n* = 9, Klatskin cancer: *n* = 7, pancreatic cancer: *n* = 6, gallbladder cancer: *n* = 5

^d^ Lymphoma: *n* = 13 (7x B-NHL, 4x CLL, 2x T-NHL), multiple myeloma: *n* = 7, leukaemia: *n* = 2 (1x ALL, 1x AML)

^e^ Endometrial cancer: *n* = 6, cervical cancer: *n* = 6, vulvar cancer: *n* = 3

^f^ Melanoma: *n* = 7, renal cell cancer: *n* = 6, CUP: *n* = 4, prostate cancer: *n* = 4, thyroid cancer: *n* = 2, mesothelioma: *n* = 1, thymic cancer: *n* = 1

Median follow-up for all patients was 1.5 months (range, 1 day-89.8 months; IQR, 0.6–4.8). At data cut-off (March 31, 2017), 20 patients were still alive (5.1%), 324 had died (82.0%), 51 were lost to follow-up (12.9%). 135 patients (34.2%) expired less than 30 days post procedure, 74 (54.8%) before discharge from hospital. Median catheter permanence was 1.2 months for all patients (range, 1 day-23.6 months; IQR, 0.5–2.6) vs. 2.1 months (IQR, 1.3–4.4; *p* < 0.001) in those surviving at least 30 days.

### Efficacy and pleurodesis

To determine the efficacy, we investigated the need of subsequent invasive procedures. Follow-up data were available for 410 procedures demonstrating no need for additional interventions in 94.9% until last visit or death. Thoracenteses or second chest tubes after IPC removal were performed 21 times (5.1%), of those 14 (3.4%) due to a preceding IPC-related complication and 7 (1.7%) after TP failure. Pleurodesis was documented in 128 procedures (28.6%) with a median onset time of 55.5 and 56 days in ovarian and lung cancer vs. 183 days in breast cancer (*p* = 0.001; remaining entities 44.5 days). An additional talc slurry pleurodesis via the IPC was successfully performed in 10 of 14 procedures (71.4%). To exclude a negative bias of patients not surviving long enough to develop pleurodesis, we further examined the subgroup of patients surviving ≥30 days (*n* = 271 patients/283 procedures). The resulting higher pleurodesis rate of 44.5% is consistent with previous findings [12]. Following pleurodesis, the catheter was removed in 105 patients (81.7%). Results for logistic regression for pleurodesis are shown in Table 2. For all procedures (*n* = 448), age < 60 years (HR 1.62, *p* = 0.024) and the use of talc slurry via the IPC (HR 6.70, *p* = 0.002) increased the likelihood of pleurodesis. The predictive effect of complications (HR 2.1, *p* = 0.002) and bilateral effusions (HR 2.52, *p* = 0.001) was no longer significant in patients surviving ≥30 days, whereas age < 60 (HR 1.71, *p* = 0.030) and talc (HR 6.68, *p* = 0.015) remained predictive. Additionally, using multiple logistic regression, both factors predicted pleurodesis independently.

**Table 2:** Univariate and multiple logistic regression analysis for (auto-) pleurodesis in all patients (448 procedures, column 1) and in patients surviving ≥30 days (283 procedures, columns 2 and 3)

Variable	Univariate logistic regression for pleurodesis, all patients (*n* = 448 procedures)			Univariate logistic regression for pleurodesis, survival ≥30 days (*n* = 283 procedures)			Multiple logistic regression for pleurodesis, survival ≥30 days (*n* = 283 procedures)		
	HR	95% CI	*p*-value	HR	95% CI	*p*-value	HR	95% CI	*p*-value
Sex									
male vs. female	0.950	0.609–1.481	0.821	0.779	0.472–1.286	0.328			
Age									
< 60 vs. ≥60	1.621	1.066–2.465	0.024^a^	1.712	1.053–2.783	0.030^a^	1.854	1.131–3.038	0.014^a^
< 65 vs. ≥65	1.169	0.776–1.762	0.455	1.143	0.715–1.827	0.577			
< 70 vs. ≥70	1.508	0.954–2.385	0.079	1.413	0.839–2.382	0.194			
< 75 vs. ≥75	1.145	0.656–2.000	0.633	1.052	0.556–1.988	0.876			
Cancer entity									
Ovarian cancer vs. other	1.379	0.897–2.120	0.143	1.397	0.852–2.291	0.185			
Breast cancer vs. Other	0.939	0.489–1.803	0.851	0.908	0.436–1.891	0.796			
Gynaecologic cancer vs. other	1.276	0.847–1.925	0.244	1.307	0.817–2.091	0.264			
Lung cancer vs. other	0.630	0.369–1.075	0.090	0.585	0.324–1.056	0.075			
Cause of effusion									
MPE vs. other	1.027	0.630–1.674	0.915	0.790	0.445–1.404	0.422			
MPE vs. paramalignant effusion	0.644	0.283–1.468	0.295	0.687	0.269–1.757	0.434			
Laterality									
Bilateral vs. unilateral	2.064	1.304–3.265	0.002^a^	1.419	0.851–2.363	0.179			
left vs. right	1.284	0.850–1.940	0.235	1.350	0.841–2.165	0.214			
Use of talc slurry via the IPC									
Yes vs. No	6.695	2.060–21.758	0.002^a^	6.681	1.436–31.075	0.015^a^	7.812	1.661–36.737	0.009^a^
Complications									
Yes vs. No	2.520	1.445–4.394	0.001^a^	1.509	0.829–2.747	0.179			

^a^*p* < 0.05

### Complications

Complications occurred in 60 procedures corresponding to 13.4% of all interventions (see Table 3), therefrom the majority concerning infections (5.6%; empyema 2.5%, infections at the exit site 2.2%, tunnel infections 0.9%) and catheter malfunctions (5.4%). Using the Clavien-Dindo classification, 59 (98.3%) were graded as mild or moderate (grade I to III). Catheter removal was performed in 23 cases (38.3% of all complications). This is considered as an intervention and therefore classified as grade III. One intervention-related death was seen in empyema following IPC-insertion, the patient subsequently developing sepsis and multi-organ failure. Median time to complication was 33.5 days (range, 0–552 days; IQR, 10–79). Dislodgements of the catheter and local infections at the exit site occurred rather early within the first 3 to 5 weeks (20.5 days (range, 3–48, IQR, 6.8–33.8) and 38 days (range, 1–186, IQR, 10.8–61)) suggesting a non-adequate adherence of the cuff to the surrounding tissue, while tunnel infections and empyema were seen significantly later (103 days (range, 8–550, IQR, 8.3–463.3) and 116 days (range 35–552 days, IQR 44–191); *p* = 0.003). Empyema were diagnosed using cultures drawn from the IPC, additional thoracenteses were not routinely performed. Simultaneous superficial and deep infections never occurred. Catheter explantation was necessary in 2 of 14 local infections (14.3%) and in 7 of 11 empyema (63.6%). All other cases were sufficiently treated with intravenous antibiotics. In empyema, microbiological cultures were positive in 81.8%, showing predominantly gram-positive cocci (*Staphylococcus aureus* 45.5%, coagulase-negative staphylococci 18.2%, viridans group streptococci 18.2%; one case each with *Bacteroides fragilis* and polymicrobial infection). All patients with empyema, except the fatal case, had a sufficient pleurodesis. Occlusion of the IPC could be restored in 10 of 13 cases (77%), either via aspiration of intraluminal fibrin clots or by applying intraluminal fibrinolysis with subsequent proper drainage. Occlusions due to symptomatic loculations were successfully dissolved with intrapleural fibrinolysis in two cases. Two IPC had to be removed since occlusion was not resolvable, in one case the catheter was left in place due to a reduced performance status. One subcutaneous emphysema occurred after insertion requiring catheter removal. Complication rates were higher in men than women (18.6 vs. 12.4% of all procedures, *p* = 0.023), attributable to a higher likelihood for mechanical catheter problems in men (8.6 vs. 3.9 of all procedures, *p* = 0.038), whereas no gender-specific differences were seen for infections, especially empyema. With regard to the institution’s standardization of handling patients with IPC (including regular appointments following IPC placement), especially infectious complications substantially decreased over time (8.6% (2006–2012) vs. 3.9% (2013–2016), *p* = 0.032).

**Table 3:** Complications

Complications	n	% (of complications)	% (of procedures)	Grade II (n)	Grade III (n)	Grade V (n)	Time to complication (days) (median, IQR)
Total	60	100.0	13.4	34	25	1	33.4 (10.3–79.3)
Aetiology							
Infections	25	41.7	5.6	16	8	1	60 (28–160)
Local infection	14	23.3	3.1	12	2	–	38 (9.8–94.5)
Cellulitis/Exit site	10	16.7	2.2	10	0	–	38 (10.8–61)
Tunnel	4	6.7	0.9	2	2	–	103 (8.3–463.3)
Deep infection (empyema)	11	18.3	2.5	4	6	1	116 (44–191)
Catheter malfunction	24	40.0	5.4	11	13	–	26 (12–62.5)
Occlusion/mechanical obstruction	13	21.7	2.9	10	3	–	27 (13.5–80.5)
Dislodgement	8	13.3	1.8	1	7	–	20.5 (6.8–33.8)
Leakage	3	5.0	0.7	–	3	–	31 (−)
Loculation	5	8.3	1.1	3	2	–	26 (4–122.5)
Bleeding	4	6.7	0.9	4	–	–	0.5 (0–4)
Subcutaneous emphysema	1	1.7	0.2	–	1	–	9 (−)
Tract metastasis	1	1.7	0.2	–	1	–	65 (−)
Catheter removed	23	38.3	5.1				

### Survival with IPC

**Fig. 1:**
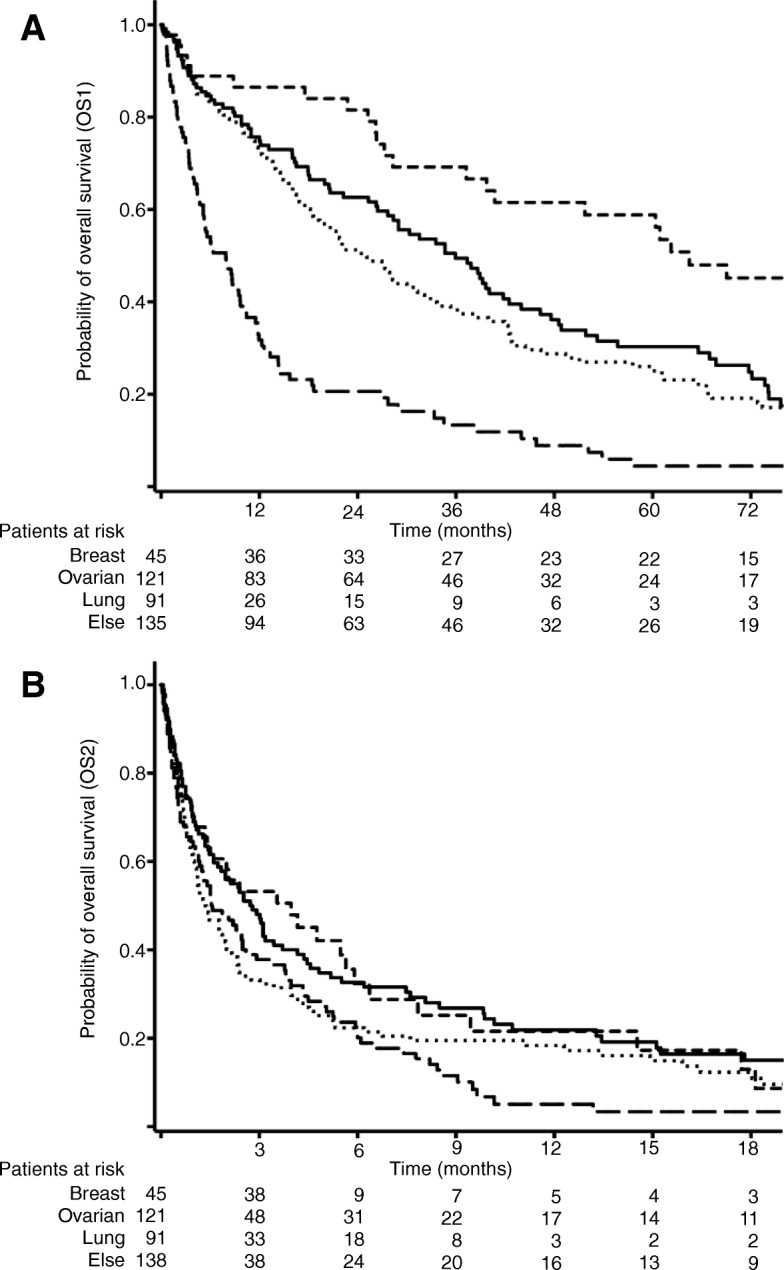
Kaplan-Meier curves for overall survival after date of primary diagnosis (OS1; **a**) and after IPC placement (OS2; **b**). Legend

For the entire cohort, median survival after diagnosis of the malignancy (OS1) was 25.3 months (95% CI, 39.5–52.3). OS1 in lung, ovarian and breast cancer were 7.9 months (95% CI, 4.6–11.2), 35.9 months (95% CI, 27.5–44.4) and 64.5 months (95% CI 36.3–92.7), respectively (*p* < 0.001, see Fig. 1: Kaplan-Meier curves for overall survival after date of primary diagnosis (OS1; A) and after IPC placement (OS2; B)). Survival after catheter insertion (OS2) reached 2.0 months in the entire cohort (95% CI, 1.5–2.4) and was 1.6 months (95% CI, 0.7–2.5), 2.8 months (95% CI, 2.0–3.6) and 4.0 months (95% CI, 1.0–7.0) in lung, ovarian and breast cancer, respectively (*p* = 0.04, see Fig. 1b). Results for uni- and multivariate Cox regression analyses with identification of prognostic factors for OS2 are shown in Table 4. Patients aged < 60 years and/or with gynaecologic malignancies had a more favourable prognosis whereas survival in lung cancer was clearly lower. Bilateral effusions also carried a better prognosis, predominantly attributed to patients with ovarian cancer. In multivariate Cox regression analysis, gynaecologic cancer and bilateral catheters were identified as independent predictors for survival.

**Table 4:** Univariate and multiple Cox regression analysis for overall survival after IPC placement (OS2)

Variable	Univariate Cox regressionfor OS2 (*n* = 395 patients)			Multivariate Cox regressionfor OS2 (*n* = 395 patients)		
	HR	95% CI	*p*-value	HR	95% CI	*p*-value
Sex						
male vs. female	1.143	0.908–1.437	0.254			
Age						
< 60 vs. ≥60	0.796	0.633–1.000	0.050^a^	0.803	0.638–1.009	0.060
< 65 vs. ≥65	0.886	0.712–1.102	0.276			
< 70 vs. ≥70	0.858	0.681–1.081	0.194			
< 75 vs. ≥75	1.046	0.773–1.415	0.769			
Cancer entity						
Gynaecologic cancer vs. other	0.753	0.604–0.940	0.012^a^	0.787	0.630–0.984	0.036^a^
Lung cancer vs. other	1.283	0.997–1.651	0.053			
Cause of effusion						
MPE vs. other	0.946	0.683–1.310	0.737			
MPE vs. paramalignant effusion	0.837	0.535–1.310	0.437			
Laterality						
Bilateral vs. unilateral	0.702	0.507–0.972	0.033^a^	0.703	0.510–0.968	0.031^a^
Bilateral vs. unilateral (Ovarian vs. else)	0.671	0.488–0.924	0.015^a^			
left vs. right	0.982	0.787–1.224	0.870			
Pleurodesis						
Yes vs. No	1.192	0.854–1.664	0.302			
Complications						
Yes vs. No	0.869	0.624–1.210	0.406			

^a^*p* < 0.05

## Discussion

Recurrent pleural effusion is frequently observed in the course of malignancy, often marking the final common pathway in many cancer patients. It is associated with a high disease burden as well as morbidity and mortality [1, 5, 7, 16]. Our study presents a large dataset of 395 patients (and 448 catheters) on clinical characteristics and outcomes with IPC in the general setting of malignancy. It is, to the best of our knowledge, the largest evaluation in gynaecologic cancer to date. These patients and those with bilateral effusions had improved survival.

The primary goal of symptom control and satisfying palliation without subsequent procedures was achieved in nearly 95% in our investigation, highlighting the efficacy of IPC in the palliative setting [23, 24]. Due to the retrospective character of our study, palliation could only be assessed in an indirect manner focusing on the need of additional (invasive) procedures like thoracenteses or chest tubes. Although this fact may overestimate the effect of IPC – as clinical variables like dyspnoea or chest pain may persist despite an adequately working drainage system – the TIME2 trial has shown comparable results [18]. Ovarian cancer, especially in advanced stages, regularly goes along with (large amounts of) ascites. In our investigation, patients with locally advanced (FIGO stage III) or metastatic ovarian cancer at primary diagnosis (FIGO stage IV) represented 92.6% of all patients. Only 1.6% had stage IA or B. Hence, > 95% also had ascites at the time of primary diagnosis, the number increased to 100% at the time of IPC implantation. Nonetheless, no patient received an abdominal indwelling catheter, emphasizing the importance to control pleural effusion for patient’s comfort. As a major contribution to pleural effusion in ovarian cancer is mediated via transdiaphragmatic pleuroperitoneal communications [25], a durable symptom relief might be achieved using an IPC only.

Pleurodesis is a convenient side effect in IPC. The reported rates widely vary from 10 to nearly 80% with a median pleurodesis rate of 45.6% in a meta-analysis [12]. In our cohort, overall pleurodesis rate was lower (28.6%), although in those patients surviving longer than 30 days after catheter implantation, we achieved a comparable pleurodesis rate of 44.5%. An aggressive daily drainage strategy has been shown to be superior to longer drainage intervals in the ASAP trial, raising the probability for pleurodesis from 24 to 47% (*p* = 0.003) [26]. The exact way how IPC lead to pleurodesis is unclear, however mechanical irritation causing local inflammation likely contributes to it [27]. As the inflammatory response diminishes with increasing age, younger patients potentially might experience pleurodesis more often. However, as age distribution in our study had a wide range (15–92 years), this question warrants further well-directed investigation. In contrast, patients in our cohort with lung cancer had a (non-significantly) lower rate of pleurodesis. Close contact of the two pleural layers is a crucial requirement for pleurodesis to occur and is therefore less likely if the adjacent lung tissue is altered, as it is often the case in lung cancer. Proof of an expandable lung was an inclusion criterion in the prospective IPC-PLUS trial, investigating the benefit of an additional TP via the IPC. The combination of both modalities doubled pleurodesis rates without significant additional adverse events and might therefore be a promising option for selected patients [21]. In our study, the 14 patients treated with an additional TP via the IPC experienced a high rate of pleurodesis as well (71.4%). Use of talc slurry was highly predictive for pleurodesis (HR 7.8, *p* = 0.009).

Survival after primary diagnosis (OS1) strongly depends on the cancer entity reflecting tumour biology as well as effective treatments available. Not surprisingly, we observed wide differences, with lung and breast cancer patients on the extremities (7.9 vs. 64.5 months). In contrast, survival after diagnosis of MPE is poor and closely correlates with the extent of the underlying disease [28–31]. The rather short survival after IPC placement (OS2) may be strongly influenced by a large proportion of patients having received the IPC as a palliative measure for symptom control with limited systemic treatment options available for cancer control. In our cohort, 34.2% of all patients died within 1 month, confirming similar rates reported in the literature [32]. Even in patients with a very limited life expectancy, IPC offer the advantage of an earlier discharge from hospital, if there is a wish for an ambulatory palliative care setting by the individual patient. Unexpectedly, in our cohort, bilateral effusions were a favourable prognostic factor in terms of survival, in contrast to most of the published literature reporting a decreased survival [32]. The high percentage of patients with ovarian cancer who regularly suffer from bilateral effusion [4] is likely to have influenced our results, though prognostic differences between uni- and bilateral pleural effusion have not been described yet [33]. Bilateral effusions were not exclusively correlated to ovarian cancer but the proportion of patients with bilateral IPC was substantially higher (19.8% in ovarian cancer vs. 6.6% in lung and 13.3% in breast cancer). Reasons for the observed survival differences remain speculative. The issue that all patients with ovarian cancer underwent an aggressive abdominal cytoreductive surgery, conferring a well-known prognostic effect, might have contributed to bilateral effusions [25].

Periprocedural and long-time complications of IPC occur in 10–15% of all patients, which conversely demonstrates that 85–90% do not suffer from any adverse event [12]. The most common ones are infections and mechanical catheter problems. The rates of pleural infections in published series with > 100 patients are generally low, ranging from 1 to 6% [11, 14, 34–38], in line with our results of 2.5%. As the most serious infectious complication, empyema typically occurs at least 6 weeks after catheter insertion, direct contamination of the pleura with bacteria during the procedure seems unlikely [15, 35]. The period of time for development of empyema reported in the literature widely varies from 5 weeks up to 3 months [35, 39, 40]. With the reported 3.8 months, empyema occurred rather late in our series. Preventive measures like patient education in handling the catheter properly therefore are the backbone of a well working IPC system. Furthermore, ambulatory care provided by specialized nursing teams may also reduce complications. In this connection, we were able to demonstrate an impressing drop of infections over the reported decade. Thus, empyema is a much feared but rare complication with low mortality rates. The majority of these cases can be treated with antibiotics, without explantation of the IPC system. As rapid evacuation of infected effusions is an important component of therapy, retaining the catheter in situ should be thoroughly considered [35]. Moreover, successfully treated empyema often result in sufficient and lasting pleurodesis. Whereas fibrin deposition leading to pleurodesis is a desired side effect of IPC, insufficiently evacuated pleural effusion may lead to loculated effusions affecting 5–14% of the patients [17, 18, 37, 41]. Similar to pleurodesis, loculation is preceded by a decreasing amount of drained fluid. Attempts to dissolve loculation using intrapleural fibrinolytics have regularly been made in the past (also in our cohort). The recently published TIME3 trial has shown the futility of intrapleural urokinase for nondraining malignant pleural effusion with regard to dyspnoea or pleurodesis. Interestingly, patients in the urokinase group experienced a shorter hospital stay and had improved survival [42]. Rates of mechanical complications in our study were higher in male patients. As the majority were due to occlusions, one might imply a certain carelessness in male patients. On the other hand, the predominance of thoracic malignancies in men (46.2 vs. 7.9%, *p* < 0.001) implicating certain anatomic particularities favouring obstructions could also have contributed to these gender-specific differences. E. g., the rate of postobstructive effusions was higher in men (10.0 vs. 4.5%, *p* = 0.04).

Due to the retrospective character, the present study has its limitations. On the one hand, the data give a close insight into management and follow-up of patients with IPC with a low rate of lost to follow-up patients. On the other hand, indication for an IPC is at least partially subject to the physician’s discretion and thereby source of selection bias. Moreover, estimation of procedure-related complications may be too low, as some events may have been undocumented in those lost to follow-up. Further, performance status as a relevant prognostic factor was not documented routinely, thereby potentially biasing survival among the different entities. Finally, our cohort may not be representative in the general setting of malignancy-related pleural effusion with the investigational site being part of the European Competence Centre for Ovarian Cancer, thereby reflecting a certain referral bias with the predominance of gynaecologic cancer.

## Conclusion

The current investigation provides a large single-centre case series with IPC in malignant diseases with a strong focus on underrepresented gynaecologic cancer in this setting until now. Our study strengthens the estimation of IPC as a feasible first-line option in the management of recurrent pleural effusion – efficient in symptom relief and with a favourable safety profile in daily routine. The observed higher rates of mechanical complications in men as compared to women warrant further investigation. With an appropriate patient education and the help of specialized nursing teams, rates of infectious complications are low, even in a long-term setting.

## Data Availability

The datasets (IBM SPSS version 24) used and/or analysed during the current study are available from the corresponding author on reasonable request.

## Abbreviations

HR: Hazard ratio
IPC: Indwelling pleural catheter
IQR: Interquartile range
MPE: Malignant pleural effusion
TP: Talc pleurodesis

## References

[1] Rodriguez-Panadero F, Borderas Naranjo F, Lopez Mejias J (1989). Pleural metastatic tumours and effusions. Frequency and pathogenic mechanisms in a post-mortem series. Eur Respir J.

[2] Hirata T, Yonemori K, Hirakawa A, Shimizu C, Tamura K, Ando M, Katsumata N, Tanimoto M, Fujiwara Y (2011). Efficacy of pleurodesis for malignant pleural effusions in breast cancer patients. Eur Respir J.

[3] Porcel JM, Gasol A, Bielsa S, Civit C, Light RW, Salud A (2015). Clinical features and survival of lung cancer patients with pleural effusions. Respirology.

[4] Wimberger P, Wehling M, Lehmann N, Kimmig R, Schmalfeldt B, Burges A, Harter P, Pfisterer J, du Bois A (2010). Influence of residual tumor on outcome in ovarian cancer patients with FIGO stage IV disease: an exploratory analysis of the AGO-OVAR (Arbeitsgemeinschaft Gynaekologische Onkologie ovarian Cancer study group). Ann Surg Oncol.

[5] American Thoracic Society (2000). Management of malignant pleural effusions. Am J Respir Crit Care Med.

[6] Sahn SA (1988). State of the art. The pleura. Am Rev Respir Dis.

[7] Roberts ME, Neville E, Berrisford RG, Antunes G, Ali NJ (2010). Group BTSPDG: Management of a malignant pleural effusion: British Thoracic Society Pleural Disease Guideline 2010. Thorax.

[8] Dresler CM, Olak J, Herndon JE, Richards WG, Scalzetti E, Fleishman SB, Kernstine KH, Demmy T, Jablons DM, Kohman L (2005). Phase III intergroup study of talc poudrage vs talc slurry sclerosis for malignant pleural effusion. Chest.

[9] Kennedy L, Rusch VW, Strange C, Ginsberg RJ, Sahn SA (1994). Pleurodesis using talc slurry. Chest.

[10] Walker-Renard PB, Vaughan LM, Sahn SA (1994). Chemical pleurodesis for malignant pleural effusions. Ann Intern Med.

[11] Putnam JB, Walsh GL, Swisher SG, Roth JA, Suell DM, Vaporciyan AA, Smythe WR, Merriman KW, DeFord LL (2000). Outpatient management of malignant pleural effusion by a chronic indwelling pleural catheter. Ann Thorac Surg.

[12] Van Meter ME, McKee KY, Kohlwes RJ (2011). Efficacy and safety of tunneled pleural catheters in adults with malignant pleural effusions: a systematic review. J Gen Intern Med.

[13] Clive AO, Jones HE, Bhatnagar R, Preston NJ, Maskell N (2016). Interventions for the management of malignant pleural effusions: a network meta-analysis. Cochrane Database Syst Rev.

[14] Mekhaiel E, Kashyap R, Mullon JJ, Maldonado F (2013). Infections associated with tunnelled indwelling pleural catheters in patients undergoing chemotherapy. J Bronchology Interv Pulmonol.

[15] Lui MM, Thomas R, Lee YC (2016). Complications of indwelling pleural catheter use and their management. BMJ Open Respir Res.

[16] Desai NR, Lee HJ (2017). Diagnosis and management of malignant pleural effusions: state of the art in 2017. J Thorac Dis.

[17] Fysh ETH, Waterer GW, Kendall PA, Bremner PR, Dina S, Geelhoed E, McCarney K, Morey S, Millward M, Musk AWB (2012). Indwelling pleural catheters reduce inpatient days over pleurodesis for malignant pleural effusion. Chest.

[18] Davies HE, Mishra EK, Kahan BC, Wrightson JM, Stanton AE, Guhan A, Davies CW, Grayez J, Harrison R, Prasad A (2012). Effect of an indwelling pleural catheter vs chest tube and talc pleurodesis for relieving dyspnea in patients with malignant pleural effusion: the TIME2 randomized controlled trial. JAMA.

[19] Boshuizen RC, Vd Noort V, Burgers JA, Herder GJM, Hashemi SMS, Hiltermann TJN, Kunst PW, Stigt JA, van den Heuvel MM (2017). A randomized controlled trial comparing indwelling pleural catheters with talc pleurodesis (NVALT-14). Lung Cancer.

[20] Thomas R, Fysh ETH, Smith NA, Lee P, Kwan BCH, Yap E, Horwood FC, Piccolo F, Lam DCL, Garske LA (2017). Effect of an indwelling pleural catheter vs talc Pleurodesis on hospitalization days in patients with malignant pleural effusion: the AMPLE randomized clinical trial. JAMA.

[21] Bhatnagar R, Keenan EK, Morley AJ, Kahan BC, Stanton AE, Haris M, Harrison RN, Mustafa RA, Bishop LJ, Ahmed L (2018). Outpatient talc administration by indwelling pleural catheter for malignant effusion. N Engl J Med.

[22] Dindo D, Demartines N, Clavien PA (2004). Classification of surgical complications: a new proposal with evaluation in a cohort of 6336 patients and results of a survey. Ann Surg.

[23] Freeman RK, Ascioti AJ, Mahidhara RS (2013). A propensity-matched comparison of Pleurodesis or tunneled pleural catheter in patients undergoing diagnostic Thoracoscopy for malignancy. Ann Thorac Surg.

[24] Suzuki K, Servais EL, Rizk NP, Solomon SB, Sima CS, Park BJ, Kachala SS, Zlobinsky M, Rusch VW, Adusumilli PS (2011). Palliation and pleurodesis in malignant pleural effusion: the role for tunneled pleural catheters. J Thorac Oncol.

[25] Porcel JM, Diaz JP, Chi DS (2012). Clinical implications of pleural effusions in ovarian cancer. Respirology.

[26] Wahidi MM, Reddy C, Yarmus L, Feller-Kopman D, Musani A, Shepherd RW, Lee H, Bechara R, Lamb C, Shofer S (2017). Randomized trial of pleural fluid drainage frequency in patients with malignant pleural effusions. The ASAP trial. Am J Respir Crit Care Med.

[27] Mercer RM, Hassan M, Rahman NM (2018). The role of pleurodesis in respiratory diseases. Expert Rev Respir Med.

[28] Bielsa S, Salud A, Martinez M, Esquerda A, Martin A, Rodriguez-Panadero F, Porcel JM (2008). Prognostic significance of pleural fluid data in patients with malignant effusion. Eur J Intern Med.

[29] Clive AO, Kahan BC, Hooper CE, Bhatnagar R, Morley AJ, Zahan-Evans N, Bintcliffe OJ, Boshuizen RC, Fysh ET, Tobin CL (2014). Predicting survival in malignant pleural effusion: development and validation of the LENT prognostic score. Thorax.

[30] Ozyurtkan MO, Balci AE, Cakmak M (2010). Predictors of mortality within three months in the patients with malignant pleural effusion. Eur J Intern Med.

[31] Zamboni MM, da Silva CT, Baretta R, Cunha ET, Cardoso GP (2015). Important prognostic factors for survival in patients with malignant pleural effusion. BMC Pulm Med.

[32] DeBiasi EM, Pisani MA, Murphy TE, Araujo K, Kookoolis A, Argento AC, Puchalski J (2015). Mortality among patients with pleural effusion undergoing thoracentesis. Eur Respir J.

[33] Vergote I, Trope CG, Amant F, Kristensen GB, Ehlen T, Johnson N, Verheijen RH, van der Burg ME, Lacave AJ, Panici PB (2010). Neoadjuvant chemotherapy or primary surgery in stage IIIC or IV ovarian cancer. N Engl J Med.

[34] Bibby AC, Clive AO, Slade GC, Morley AJ, Fallon J, Psallidas I, Pepperell JCT, Slade MG, Stanton AE, Rahman NM (2015). Survival in patients with malignant pleural effusions who developed pleural infection: a retrospective case review from six UK centers. Chest.

[35] Fysh ETH, Tremblay A, Feller-Kopman D, Mishra EK, Slade M, Garske L, Clive AO, Lamb C, Boshuizen R, Ng BJ (2013). Clinical outcomes of indwelling pleural catheter-related pleural infections: an international multicenter study. Chest.

[36] Ost DE, Jimenez CA, Lei X, Cantor SB, Grosu HB, Lazarus DR, Faiz SA, Bashoura L, Shannon VR, Balachandran D (2014). Quality-adjusted survival following treatment of malignant pleural effusions with indwelling pleural catheters. Chest.

[37] Tremblay A, Michaud G (2006). Single-center experience with 250 tunnelled pleural catheter insertions for malignant pleural effusion. Chest.

[38] Warren WH, Kalimi R, Khodadadian LM, Kim AW (2008). Management of malignant pleural effusions using the Pleur(x) catheter. Ann Thorac Surg.

[39] Faiz SA, Pathania P, Song J, Li L, Balachandran DD, Ost DE, Morice RC, Shannon VR, Bashoura L, Eapen GA (2017). Indwelling pleural catheters for patients with hematologic malignancies. A 14-year, single-center experience. Ann Am Thorac Soc.

[40] Chalhoub M, Harris K, Castellano M, Maroun R, Bourjeily G (2011). The use of the PleurX catheter in the management of non-malignant pleural effusions. Chron Respir Dis.

[41] Thomas R, Piccolo F, Miller D, MacEachern PR, Chee AC, Huseini T, Yarmus L, Bhatnagar R, Lee HJ, Feller-Kopman D (2015). Intrapleural fibrinolysis for the treatment of indwelling pleural catheter-related symptomatic Loculations: a multicenter observational study. Chest.

[42] Mishra EK, Clive AO, Wills GH, Davies HE, Stanton AE, Al-Aloul M, Hart-Thomas A, Pepperell J, Evison M, Saba T (2018). Randomized controlled trial of Urokinase versus placebo for nondraining malignant pleural effusion. Am J Respir Crit Care Med.

